# Why language matters: a tour through hand hygiene literature

**DOI:** 10.1186/s13756-017-0218-8

**Published:** 2017-06-14

**Authors:** Daniela Pires, Ermira Tartari, Fernando Bellissimo-Rodrigues, Didier Pittet

**Affiliations:** 1 Infection Control Programme and WHO Collaborating Centre on Patient Safety - Infection Control & Improving Practices, University of Geneva Hospitals and Faculty of Medicine, 4 Rue Gabrielle-Perret-Gentil, 1211 Geneva, Switzerland; 2 Department of Infectious Diseases, Centro Hospitalar Lisboa Norte and Faculdade de Medicina da Universidade de Lisboa, Lisbon, Portugal; 3 Infection Control Unit, Mater dei Hospital, Msida, Malta; 4 Social Medicine Department, Ribeirão Preto Medical School, University of São Paulo, Ribeirão Preto, São Paulo, Brazil

**Keywords:** Hand hygiene, Hand disinfection, Hand sanitizers, Alcohol-based hand rub, Hand rubbing, Healthcare-associated infection, Infection prevention

## Abstract

**Background:**

Hand hygiene has evolved over the last decades and many terminologies emerged. We aimed to analyse the evolution in the frequency of utilization of key hand hygiene terms in the literature along the years.

**Methods:**

We identified keywords and Medical Subject Headings (MeSH) used in MEDLINE® indexation related to hand hygiene by searching international guidelines and the MeSH database. We performed a MEDLINE® search combining the selected keywords and MeSH and analysed the number of publications retrieved yearly.

**Results:**

The literature search yielded 9019 publications when all hand hygiene related search terms were combined, between 1921 and November 2016. The total number of publications per year increased from a median of 4 (IQR 3, 6) in the 1950’s to 554 (IQR 478, 583) between 2011 and 2015. The most frequently used keywords are hand disinfection, hand hygiene, hand washing, handrub, hand sanitizer and alcohol-based hand rub (ABHR). Until the 1990s, hand disinfection and hand washing were the most frequently used terms. Whilst the last decade has seen a remarkable increase in publications mentioning hand disinfection and hand hygiene and for the first time handrub, hand sanitizers and ABHR were introduced in the literature. Hand disinfection, hand hygiene and hand sanitizers are the main MeSH used by MEDLINE®. Since 2013 hand hygiene is the most frequently used MeSH and keyword.

**Conclusions:**

The change seen in literature in the last two decades, from hand washing and hand disinfection to hand hygiene, most probably reflect the paradigm shift favouring use of ABHR over soap and water promoted by international guidelines in the early 2000s.

## Background

Exactly 170 years ago Ignas Semmelweis demonstrated the first evidence that cleansing heavily contaminated hands with an antiseptic solution reduced infections and provided safer obstetric care [1]. However, hand washing with soap and water remained the standard of practices in healthcare until the late 1990s. In the turning of the millennium, the Centers for Disease Control and Prevention (CDC) [2] and the World Health Organization (WHO) [3, 4] hand hygiene guidelines have shaped the evolution in this field and defined alcohol-based hand rub (ABHR) as the new standard of care in healthcare settings worldwide [2, 3]. Interestingly, many hand hygiene related terminologies have emerged in literature. We aimed to explore the evolution of key terms used in hand hygiene literature in the last decades thought the analysis of the number of publications retrieved in MEDLINE® and related it to the evolution in the field itself.

## Methods

We identified hand hygiene related keywords by searching the CDC and WHO hand hygiene guidelines. The keywords were selected by author’s consensus. Additionally, we identified Medical Subject Headings (MeSH) used by MEDLINE® to index hand hygiene literature by searching the MeSH database. In November 2016 we searched MEDLINE® with the selected keywords and MeSH terms to obtain the total number of hand hygiene-related publications. Subsequently, we analysed the evolution of the number of publications over time.

## Results

**Fig. 1:**
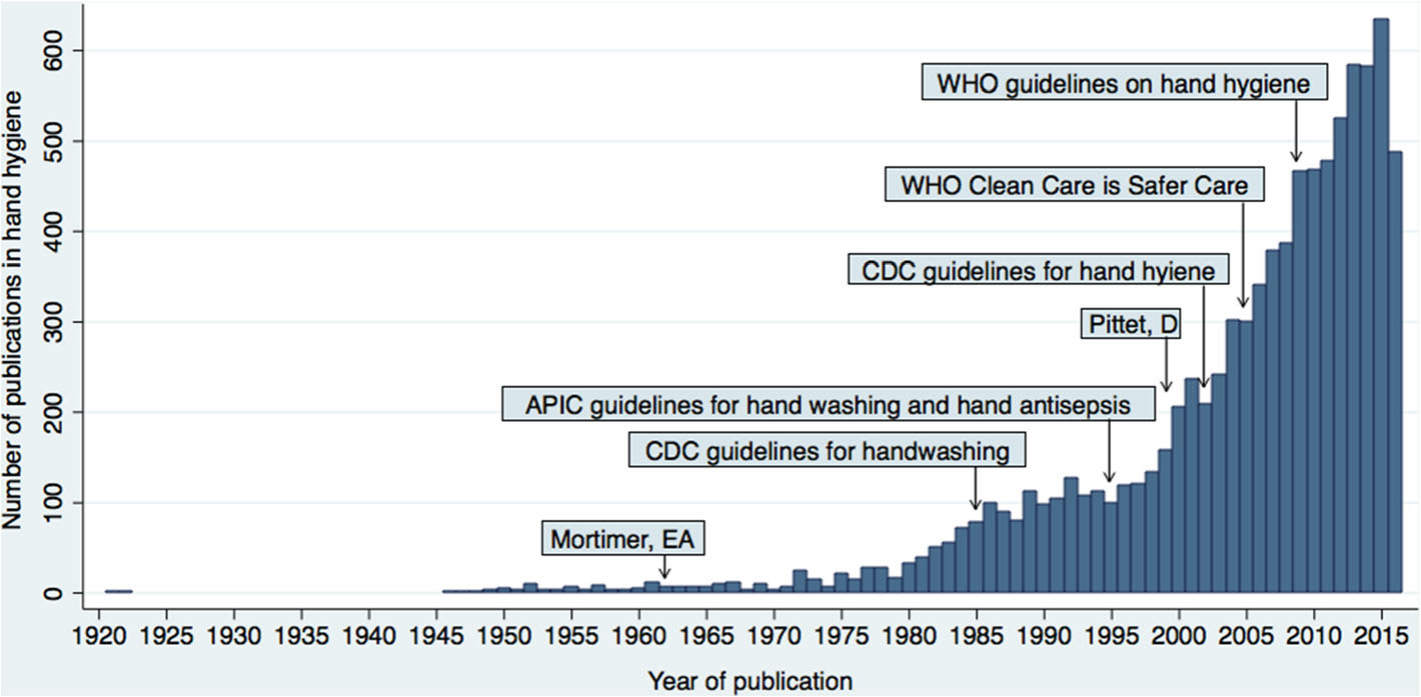
Number of publications on hand hygiene retrieved in MEDLINE® by year. The search was conducted on the 3 of November 2016 according to the search strategy described in Table 1 under “all keywords”. APIC: Association for Professionals in Infection Control and Epidemiology; CDC: Centers for Disease Control and Prevention; WHO: World Health Organization

A comprehensive search of all keywords and MeSH combined retrieved a total of 9019 publications, between 1921 and November 2016 (Fig. 1). The median number of publications per year mentioning a hand hygiene related-term was: 0 (IQR 0, 2) in the 1940’s, 4 (IQR 3, 6) in the 1950’s, 7 (IQR 6, 9) in the 1960’s, 16 (IQR 7, 25) in the 1970’s, 75 (IQR 51, 90) in the 1980’s, 116 (IQR 105, 127) in the 1990’s, 301 (IQR 237, 379) in the 2000’s and 554 (IQR 478, 583) from 2011 to 2015 (Fig. 1).

**Fig. 2:**
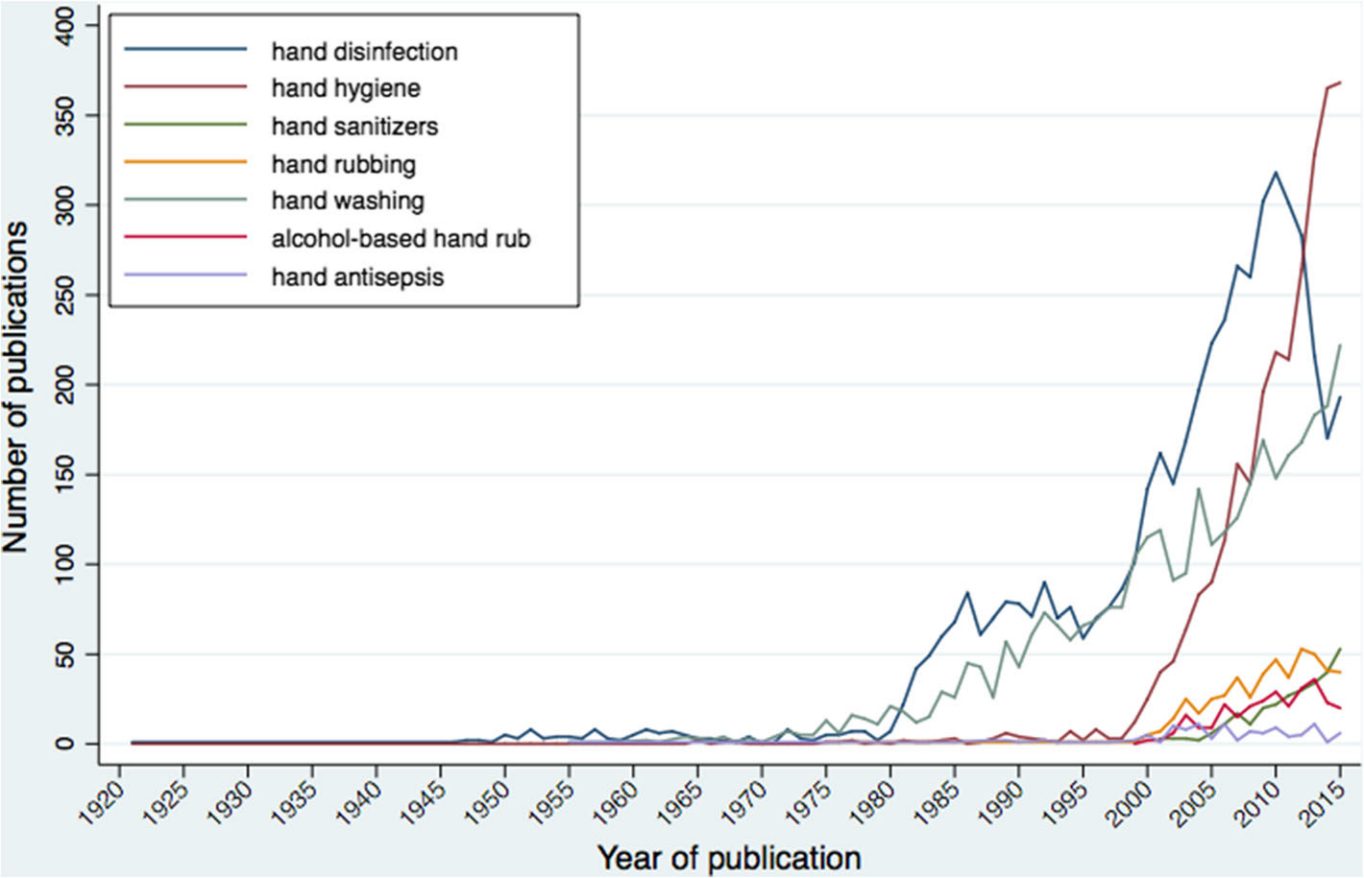
Number of publications on hand hygiene retrieved in MEDLINE® when searching the selected keywords, by year. The search was conducted on the 3 of November 2016 according to the search strategy described in Table 1 for each selected keyword

The keywords most frequently found in publications are hand disinfection (5083), hand washing (3564), hand hygiene (3085), handrub (532), hand sanitizer (317) and alcohol-based hand rub (ABHR; 306) (Table 1). However, their relative frequency of use has changed over time (Fig. 2). Until the late nineties, the great majority of articles published have referred to hand disinfection and hand washing. The term hand hygiene itself was rarely used. In the early 2000s, there was a surge of publications stating hand disinfection and hand hygiene and for the first time handrub, hand sanitizer and ABHR were introduced in the literature. Hand washing remains a frequently used term but has not paralleled the increase seen in the use of hand disinfection and hand hygiene in the turning of the millennium. The number of publications mentioning hand hygiene surpassed both that of hand washing in 2007 and hand disinfection in 2013 (Fig. 2).

**Table 1:** Summary of keywords commonly used in hand hygiene literature and number of publications retrieved by the indicated search strategy

Hand hygiene literature commonly used keywords^a^	Search strategy^b^	Number of articles	Search details^c^
hand hygiene	“hand hygiene”[Mesh]	5401	“Hand Hygiene”[Mesh]
	“hand hygiene”	3085	“hand hygiene”[All Fields]
hand disinfection	“hand disinfection”[Mesh]	4868	“hand disinfection”[Mesh]
	hand disinf*	5083	hand disinfectant[All Fields] OR hand disinfectants[All Fields] OR hand disinfection[All Fields] OR hand disinfections[All Fields]
hand sanitizers	“hand sanitizers”[Mesh]	66	“hand sanitizers”[Mesh]
	hand sanit*	317	hand sanitation[All Fields] OR hand sanitiser[All Fields] OR hand sanitisers[All Fields] OR hand sanitising[All Fields] OR hand sanitization[All Fields] OR hand sanitizer[All Fields] OR hand sanitizers[All Fields] OR hand sanitizing[All Fields]
hand washing	“hand washing “OR “handwashing “OR “hand wash“	3564	“hand washing”[All Fields] OR “handwashing”[All Fields] OR “hand wash”[All Fields]
hand rubbing	hand rub* OR handrubbing	532	(hand rub[All Fields] OR hand rubbing[All Fields] OR hand rubs[All Fields]) OR handrubbing[All Fields]
surgical scrubbing	surgical scrub*	271	surgical scrub[All Fields] OR surgical scrubbing [All Fields] OR surgical scrubs [All Fields]
hand cleansing	hand cleans*	95	hand cleanser[All Fields] OR hand cleansers [All Fields] OR hand cleansing [All Fields]
hand decontamination	hand deconta*	59	hand decontaminants[All Fields] OR hand decontaminating[All Fields] OR hand decontamination[All Fields]
hand cleaning	“hand cleaning“	34	“hand cleaning”[All Fields]
hand antisepsis	hand-antisep*	126	hand antisepsis[All Fields] OR hand antiseptic[All Fields] OR hand antiseptics[All Fields]
alcohol-based hand rub	alcohol-based hand rub*	306	alcohol based hand rub[All Fields] OR alcohol based hand rubbing[All Fields] OR alcohol based hand rubs[All Fields]
ALL keywords	ALL keywords^d^	9019	e

Search performed on the 03th of November of 2016

^a^The terms used for search resulted from a consensus between the authors

^b^The terms are stated as typed in PubMed®

^c^Refers to the content shown on the search details box on the NCBI home page after typing the specific MEDELINE® strategy stated

^d^The search strategy used was: (“Hand Hygiene”[Mesh] OR “hand hygiene” OR “hand disinfection”[Mesh] OR hand disinf* OR “hand sanitizers”[Mesh] OR hand sanit* OR “hand washing” OR “handwashing” OR “hand wash” OR hand rub* OR “handrubbing” OR hand cleans* OR hand deconta* OR “hand cleaning” OR alcohol-based hand rub* OR hand-antisep* OR surgical scrub*)

^e^The search details retrieved were: (“Hand Hygiene”[Mesh] OR “hand hygiene”[All Fields] OR “hand disinfection”[Mesh] OR (hand disinfectant[All Fields] OR hand disinfectants[All Fields] OR hand disinfection[All Fields] OR hand disinfections[All Fields]) OR “hand sanitizers”[Mesh] OR (hand sanitation[All Fields] OR hand sanitiser[All Fields] OR hand sanitisers[All Fields] OR hand sanitising[All Fields] OR hand sanitization[All Fields] OR hand sanitizer[All Fields] OR hand sanitizers[All Fields] OR hand sanitizing[All Fields]) OR “hand washing”[All Fields] OR “handwashing”[All Fields] OR “hand wash”[All Fields] OR (hand rub[All Fields] OR hand rubbing[All Fields] OR hand rubs[All Fields]) OR “handrubbing”[All Fields] OR (hand cleanser[All Fields] OR hand cleansers[All Fields] OR hand cleansing[All Fields]) OR (hand decontaminants[All Fields] OR hand decontaminating[All Fields] OR hand decontamination[All Fields]) OR “hand cleaning”[All Fields] OR (alcohol based hand rub[All Fields] OR alcohol based hand rubbing[All Fields] OR alcohol based hand rubs[All Fields]) OR (hand antisepsis[All Fields] OR hand antiseptic[All Fields] OR hand antiseptics[All Fields]) OR (surgical scrub[All Fields] OR surgical scrubbing[All Fields] OR surgical scrubs[All Fields])

**Fig. 3:**
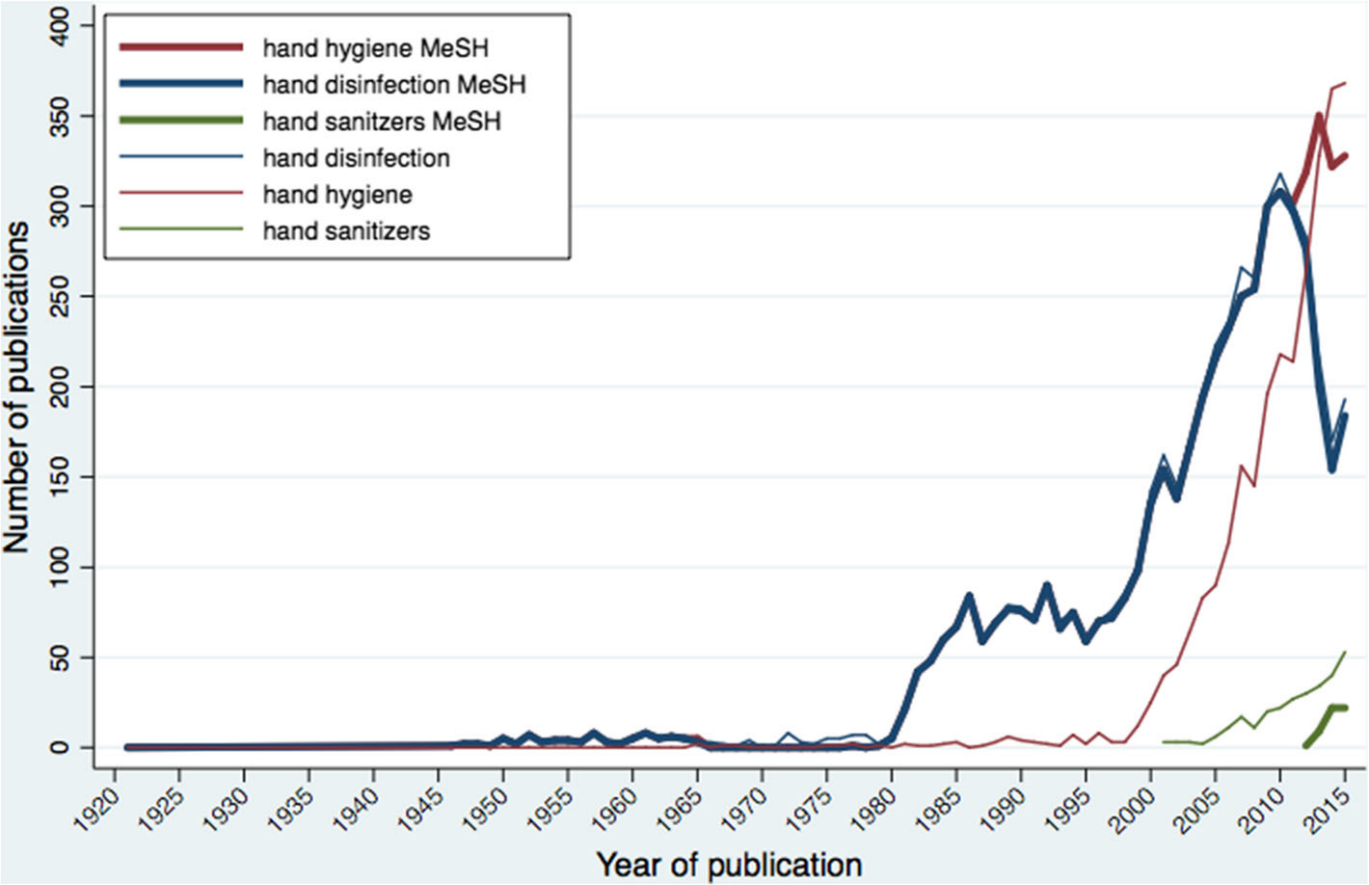
Number of publications on hand hygiene retrieved in MEDLINE® when searching the specific MeSH terms hand hygiene, hand disinfection and hand sanitizers and related keywords, by year. The search was conducted on the 3 of November 2016 according to the search strategy described in Table 1 for the specific MeSH terms and each selected keyword. MeSH: medical subject headings

The main MeSH used by MEDLINE® to index hand hygiene literature are hand disinfection (introduced in 1982), hand hygiene (2013) and hand sanitizers (2014; Fig. 3). Hand hygiene is today the most frequently used MeSH and is a broader term than hand disinfection in the MeSH tree structure. However, there are 10 entry terms for hand disinfection (such as hand washing, surgical scrubbing and hand sanitization), but none for hand hygiene (besides hand hygiene itself). Hand disinfectants and hand antiseptics are entry terms for hand sanitizers.

The keywords and MeSH that are commonly used in hand hygiene literature and the number of publications retrieved by specific search strategies in MEDLINE® are summarized in Table 1.

## Discussion

Today, the global number of scientific publications and cited references in all disciplines grows at a rate of 8-9% per year [5]. As a fair remark on the utmost importance of the main tool that guides us through this overwhelming number of publications, Smith and Chalmers have stated: “America's two greatest gifts to the world are jazz and MEDLINE” [6].

Many terminologies have emerged in hand hygiene literature over the last two decades. Some terms can be used interchangeably, but important differences remain and seem to reflect the *status quo* in the field at a given time. Briefly, hand hygiene is the general term referring to the application of soap and water or ABHR on the surface of the hands with the objective of preventing cross-transmission of pathogens in healthcare. Hand washing refers to washing hands with soap and water and has been the preferred method to perform hand hygiene until the CDC hand hygiene guidelines were issued in 2002 [2]. Hand rubbing with ABHR is the method and terminology recommended by most international hand hygiene guidelines since 2002 [2, 3]. Hand disinfection is nowadays considered a misconception since the term disinfection is only correctly applied to inanimate surfaces [3].

The importance of hand hygiene in healthcare was already recognized in the early 1960s, as it is shown by the landmark study on the prevention of *S. aureus* transmission by hand washing [7]. However, in the 1960’s only around 7 papers were issued on the topic each year. Although hand hygiene was recognized as important, it was clearly not on the research agenda. The 1980s saw an important increase in publications. Importantly, in 1985 the CDC considered hand washing with soap and water as the most important measure to prevent nosocomial infections [8]. Reflecting the standard of practices at the time, the most frequently used terms in the literature were hand disinfection and hand washing (Fig. 2).

The 1990s witnessed the beginning of a thorough implementation of Semmelweis’ hand hygiene concepts dating back to 170 years: the use of ABHR was identified as an alternative to hand washing with soap and water [9]. By this time, several studies that would establish evidence on the advantages of using ABHR, and the effectiveness of a hospital-wide multimodal strategy to improve hand hygiene, were being conducted [10, 11]. These studies served as a stepping stone for transatlantic collaboration and the publication of the CDC guidelines for Hand Hygiene (2002) that would bring the definitive paradigm shift in hand hygiene from hand washing with soap and water to handrubbing with ABHR [2]. Additionally, a significant step towards the globalization of the new hand hygiene concept was made through the launch of the WHO *‘Clean Care is Safer Care’* programme (2005) and the publication of the final WHO guidelines (2009) [3, 4, 12]. Reflecting this, the last decade appear to have achieved a surge in publications mentioning hand disinfection and hand hygiene and new terms, such as hand rubbing, alcohol-based handrub and hand sanitizers were introduced in the literature (Fig. 2). Despite hand hygiene being used very frequently as a keyword since early 2000’s, it is only in 2013 that this keyword definitively surpassed hand disinfection and that the MeSH hand hygiene is introduced by MEDLINE®. However, handrub, hand sanitizer and ABHR are still not frequently used. Importantly, handrub and ABHR are not entry terms to any of the MeSH vocabulary referred and searching MEDLINE® solely by using these keywords will miss on important publications. Meanwhile, hand washing is still used very often (Fig. 2). This could reflect a misconception regarding the preferred method and/or terminology related to hand hygiene in healthcare. However, one must keep in mind that hand washing is a broad search term, and we could be retrieving publications making reference to hand washing in the community setting, for example.

This study has limitations. We didn’t perform an analysis to ascertain the contents of the retrieved papers, i.e., to confirm that hand hygiene was a topic of research in each publication. Thus, the number of papers retrieved could also reflect other related areas of research. Furthermore, the keywords were selected by author’s consensus.

## Conclusions

The change of terminology in the literature reflects the evolution of the hand hygiene concept itself. Although the hand hygiene guidelines in the early 2000s have endorsed the term hand hygiene, it took almost a decade for hand hygiene to became the most used keyword and to be introduced as a MESH in MEDLINE®. Indeed, the addition of hand hygiene (2013) and hand sanitizers (2014) in MEDLINE® indexation has caught up with the evolution in the topic reflecting the current state of art in hand hygiene.

Although it is relatively straightforward to assume that the surge in hand hygiene publications is related to the increasing interest in this field, we cannot readily infer that this growing interest translates into better practices. Nevertheless, the growing interest given to hand hygiene as a research topic is essential in making hand hygiene an institutional and national patient safety priority.

## Abbreviations

ABHR: Alcohol-based handrub
APIC: Association for Professionals in Infection Control and Epidemiology
CDC: Centers for Disease Control and Prevention
HCW: Healthcare workers
IPC: Infection prevention and control
MeSH: Medical subject headings
NCBI: United States National Center for Biotechnology Information
NEJM: New England Journal of Medicine
SENIC: Study on the Efficacy of Nosocomial Infection Control
US: United States
WHO: World Health Organization
